# Correlation between systemic blood pressure and free flap perfusion: A photoplethysmography study

**DOI:** 10.3205/iprs000194

**Published:** 2026-04-07

**Authors:** Shamendra Anand Sahu, Jiten Kumar Mishra, Moumita De, Jalaz Joezer Rahmi, Abhijith Valsalan, Ripu Daman Arora

**Affiliations:** 1 Department of Burns & Plastic Surgery, All India Institute of Medical Sciences, Raipur, India; 2 Department of Burns & Plastic Surgery, All India Institute of Medical Sciences, Guwahati, India; 3 Department of Otolaryngology & Head Neck Surgery, All India Institute of Medical Sciences, Raipur, India

## Abstract

**Background:**

Microvascular tissue transfer is the method of choice for oncological and trauma reconstruction. Successful free tissue transfer requires technical expertise and management of patients and surgery related factors. However, whether systemic blood pressure (SBP) affects perfusion of free flaps remains unclear.

**Methods:**

Photoplethysmography (PPG) is a relatively new technology used in many wearable medical devices to measure blood pressure and heart rate. It is a simple optical technique that detects changes in blood volume within the underlying microvascular bed of tissue. This study aimed to evaluate the relationship between systemic blood pressure (SBP) and free flap perfusion using PPG.

**Results:**

Twenty-five patients who underwent free flap reconstruction for head-neck cancers or traumatic soft tissue defects were enrolled in this study. All patients were clinically normotensive without needing postoperative vasopressors. The study did not find a statistically significant correlation between systolic or mean blood pressure and free flap perfusion.

**Conclusion:**

Beyond a critical threshold of mean arterial pressure (MAP), free flap perfusion is not directly correlated with blood pressure. In clinically normovolemic patients, elevations of BP above MAP ~65 mm Hg did not enhance flap perfusion.

## Introduction

Microsurgery and super microsurgery have transformed the concept of soft tissue reconstruction from the reconstructive ladder to the reconstructive triangle. The success of free tissue transfer has increased to 99% due to improvements in instrumentation and training in microsurgery. The immediate cause of free flap failure is arterial thrombosis, but venous thrombosis is more common than arterial thrombosis [1]. A strict postoperative monitoring protocol is essential for early identification and salvage of free flaps. Many published series recommend hourly monitoring of free flaps for an initial 48–72 hours [2]. However, this is the gold standard and monitoring of the free flap by seeing bright red blood on scratch, has disadvantages including subjective variability and labor intensiveness. Thus, newer monitoring techniques are searched upon. Emerging tools and strategies include implantable Doppler, acoustic Doppler sonography, color duplex ultrasonography, flow coupler, laser Doppler flowmetry, near-infrared spectroscopy, and visible light spectroscopy [3]. Yet, none of these advances is superior to clinical monitoring of the free flap. Recently, remote photoplethysmography has been used to continuously monitor the perfusion of the free flaps intraoperatively [4]. 

In addition to surgical technique, many perioperative factors determine the success of free tissue transfer. Fluid therapy, adequate analgesia, temperature control, hematocrit, vasoactive drugs, and blood pressure are some important parameters to consider. Notably, the optimal level or range of blood pressure for maintaining adequate perfusion of the free flap is highly debated. The team hypothesised no meaningful correlation exists between systemic blood pressure (SBP) and flap photoplethysmography (PPG) within the normotensive range. So, this prospective study aimed to establish the relationship between blood pressure and perfusion of the free flap using photoplethysmography. 

## Methodology

### Patients

This study was approved by the ethical committee (IEC/2022/1043). Twenty-five patients who underwent free flap reconstruction in head-neck cancers and soft tissue defects in trauma cases were included. Cases in which a part of skin paddle of the flap was available to attach the probe were included for Photoplethysmography (PPG). Patients with chronic comorbidities such as hypertension were excluded from the study. PPG was performed only in patients who were clinically normovolemic (clinically stable patient with pulse rate between 60–100/minute, blood pressure as per age with no orthostatic hypotension, urine output ≥0.5–1.0 ml/kg/hr and capillary filling time ≤3 seconds) without the need for postoperative vasopressors. 

### Technique

**Figure 1:**
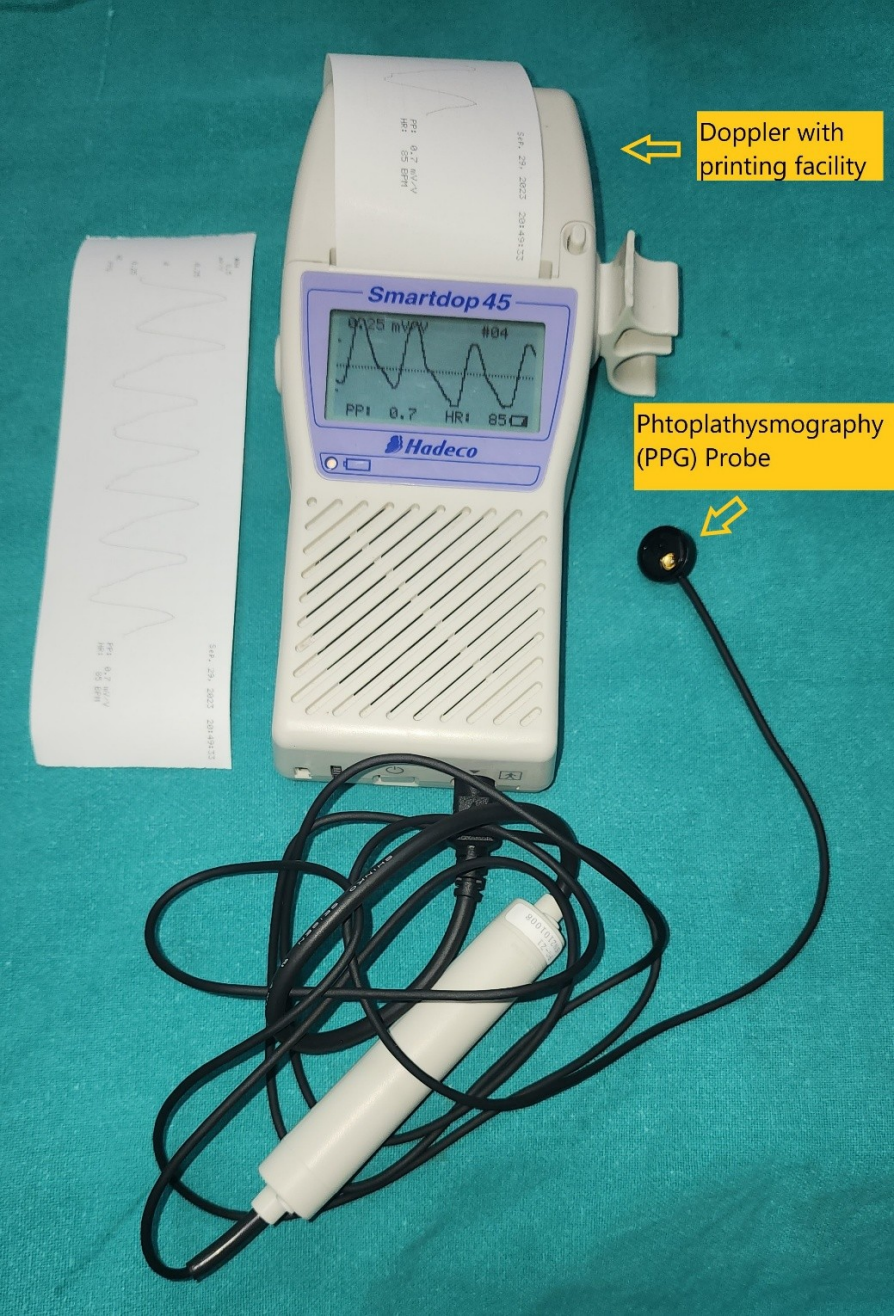
Hand held Doppler with printing facility, photoplethysmography probe and sensor

**Figure 2:**
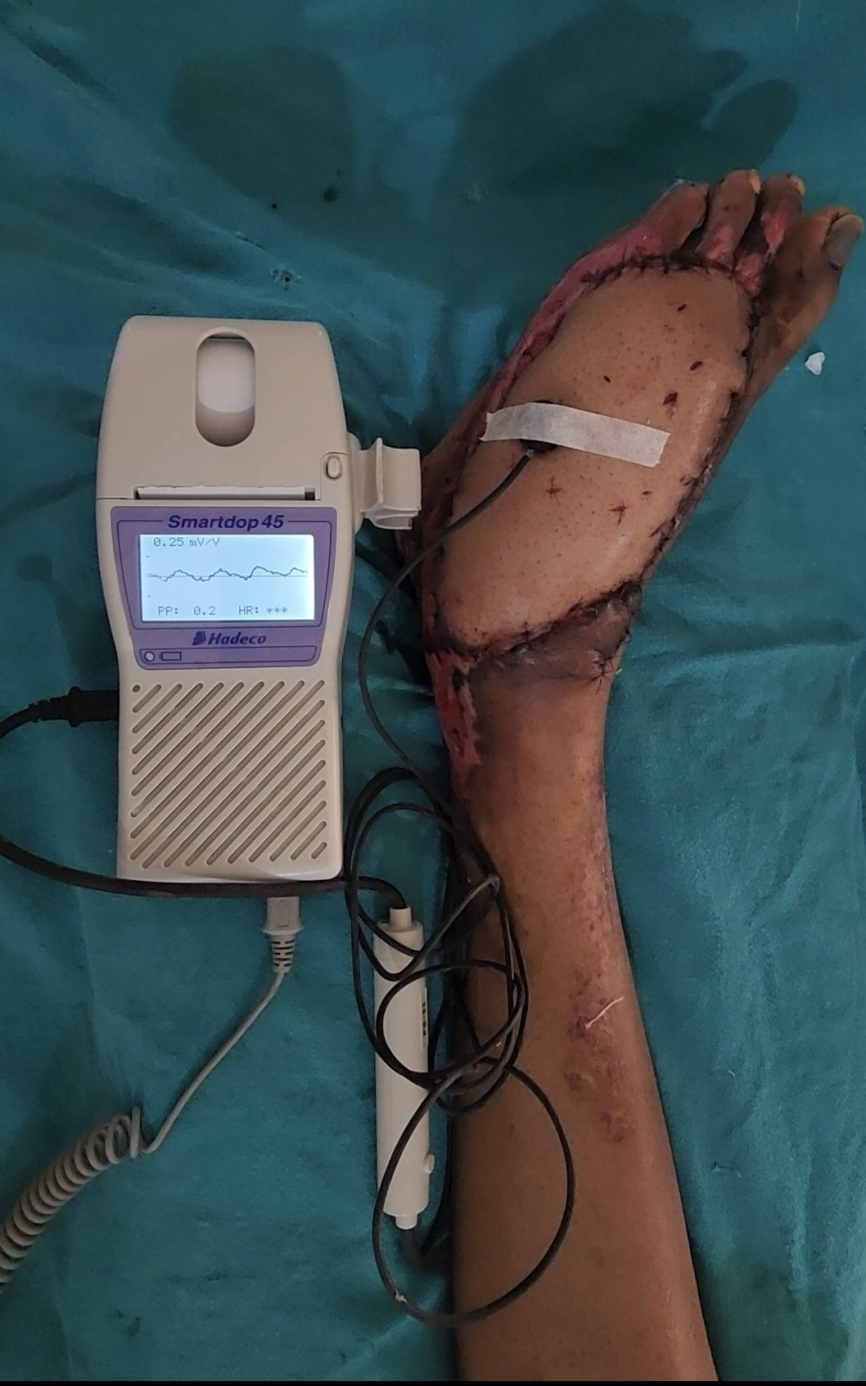
Photoplethysmography sensor secured to free flap to take reading

**Figure 3:**
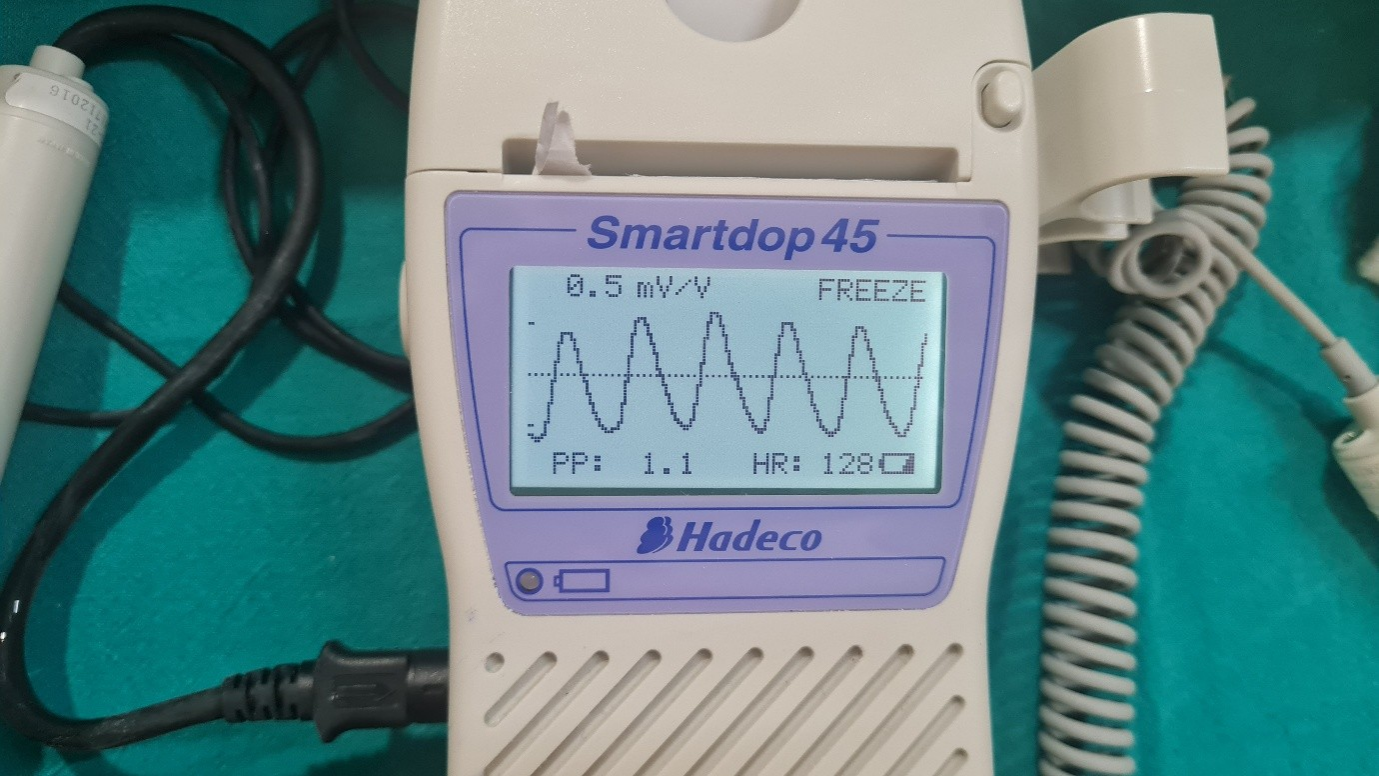
Appearance of photoplethysmography waveform in Doppler

**Figure 4:**
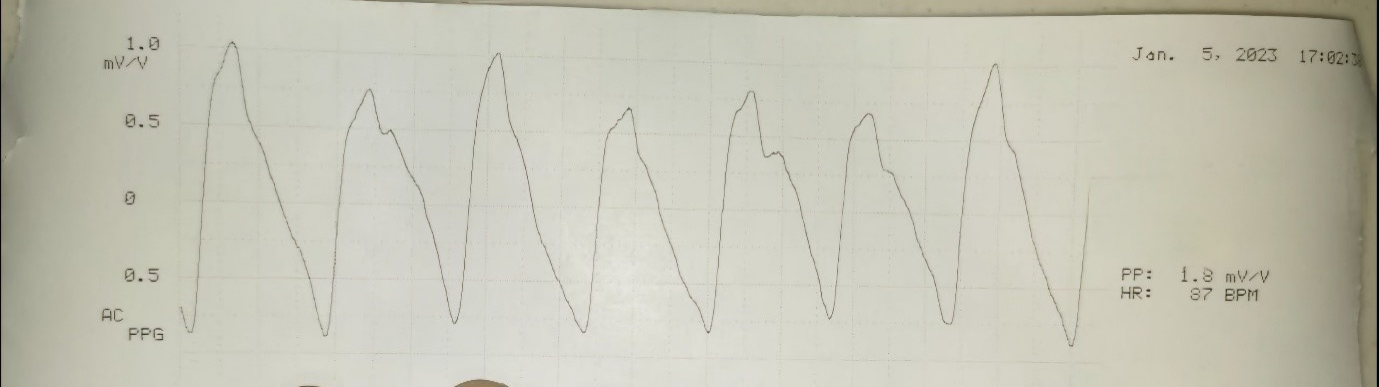
Printout of wave from for record keeping

PPG was performed using a reflectance optical probe connected to a Smartdop 45 device (manufactured by Hadeco Inc.), which includes an integrated printing facility (Figure 1 (Fig. 1)). The device had an additional 8-MHz handheld Doppler probe that can be used to locate the skin perforator. The indoor ambient temperature was maintained between 22–26°C which keeps the patients comfortable as cold temperature constricts superficial blood vessels and therefore jeopardizing the accuracy of PPG. Before placing the PPG sensor on the flap, the probe surface was cleaned using spirit. The probe was secured to the flap using micropore tape (Figure 2 (Fig. 2)). After 5–6 seconds, the waveform appeared on the monitor, and when it stabilized and became rhythmic, the probe button was pressed to freeze the waveform. A printout was taken for record keeping (Figure 3 (Fig. 3), Figure 4 (Fig. 4)). 

**Figure 5:**
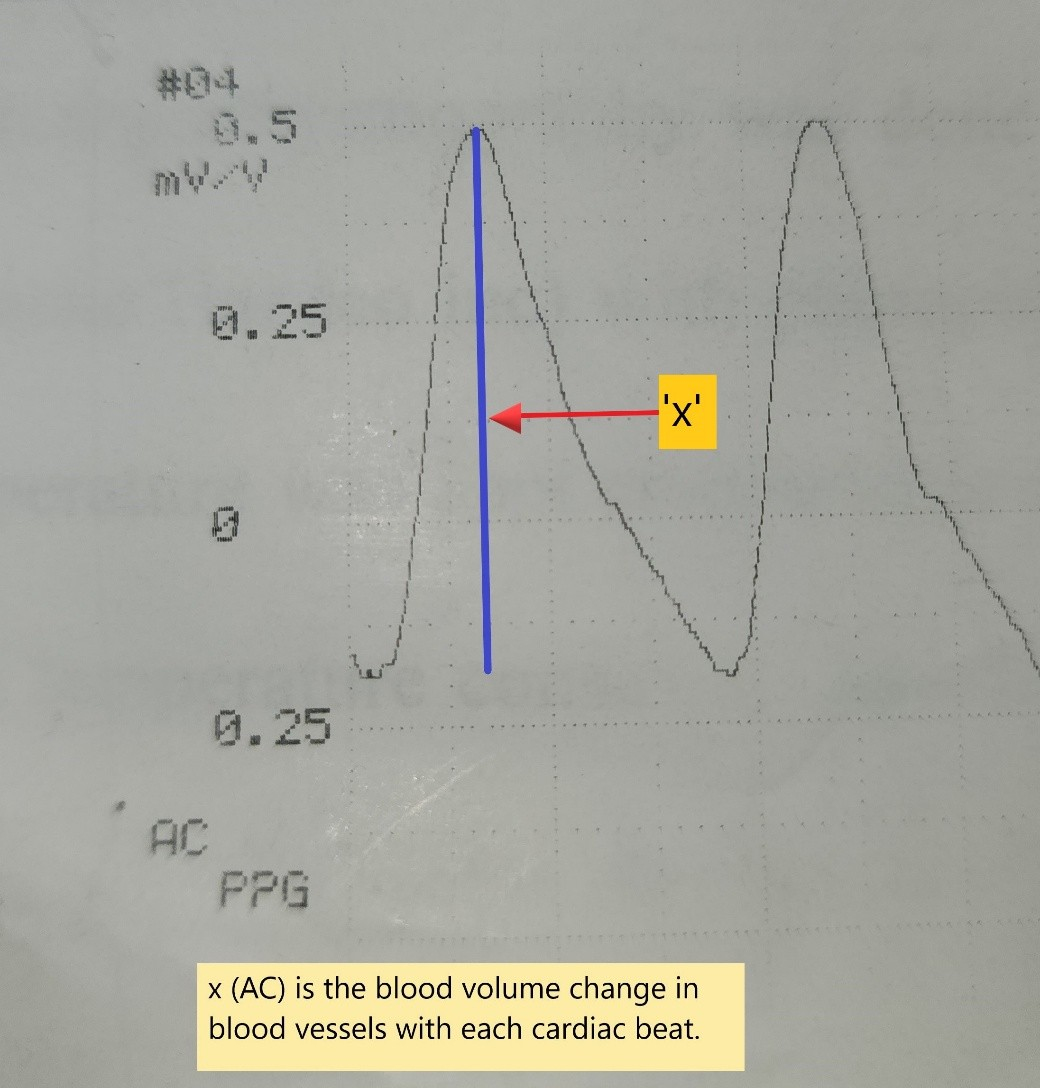
Photoplethysmography signal

The waveform signal comprises a pulsatile component (AC) and a relatively slow-varying component (DC). The systolic amplitude (AC) which is shown as ‘x’ in the figure is an indicator of the pulsatile changes caused by arterial blood flow beneath the measurement site (Figure 5 (Fig. 5)). This amplitude corresponds to the amount of blood pushed into the flap with each beat. The DC represents the constant absorption of non-pulsatile blood in the light path including venous blood, venules, and non-pulsatile arterial blood [5], [6]. The amplitude AC of the waveform is measured in mV/V units as written in the printout of the waveform. Systemic blood pressure was simultaneously recorded from the multiparameter monitor in mm Hg units. Simultaneous photoplethysmography (PPG) and blood pressure measurements were taken every 8 hours from postoperative days 1 to 3, starting at approximately 06:00, 14:00, and 22:00 hours. The data were entered in an individual case record form. 

For each patient, the mean of three readings of SBP, DBP (diastolic blood pressure), MAP, and flap PPG was calculated and used as the representative value for that day. Subsequently, daily mean values for days 1, 2, and 3 were entered into the master Excel sheet. The overall mean for each parameter on days 1, 2, and 3 was then derived from the individual patient means. 

### Data analysis

Schrey et al. reported a correlation coefficient (*r*=0.63) between blood pressure and the partial pressure of tissue oxygen in free flaps among patients with uncompromised flaps [7]. With this reported correlation of 0.63, alpha error of 5%, power of 80% with confidence interval of 95%, the sample size calculated was 24 for the study. A total of 25 patients were included in the study. Cumulative mean SBP, MAP, and flap PPG value of all patients on days 1, 2, and 3 were calculated (Table 1 (Tab. 1)). The overall mean value of 3 days was also calculated. To calculate the association between values of SBP/MAP and PPG, the Pearson correlation coefficient (*r*) was calculated for each day. Using *r*, the *p*-value of the association between the mean SBP/MAP and PPG of day 1, day 2 and day 3 was calculated. The overall *p*-value over all 3 days was also calculated (Table 2 (Tab. 2)). The overall Spearman rank correlation coefficients (r*_s_*) between cumulative PPG and SBP/MAP were also calculated. Statistical analysis was performed using SPSS for Windows. Continuous and categorical variables were expressed as mean ± SD and percentages, respectively. Pearson’s correlation test was applied to calculate the association between the two variables. Two-sided *p*-values were considered statistically significant if *p*<0.05. 

## Results

The patients included in the study were aged between 18 and 63 years. Of these, 25 were male and 10 were female. Details of the free flaps and their indications are presented in Table 3 (Tab. 3). All free flaps survived without re-exploration. The statistical analysis showed no correlation between systolic blood pressure/mean blood pressure and perfusion of the free flaps as indicated by the PPG value on day 1, 2, and 3 and for the overall value for all 3 days. The overall mean MAP 91.26±5.83 mm Hg; mean PPG 1.65 ± 0.76 mV/V; overall MAP-PPG ‘r’(Pearson’s Correlation coefficient) =0.206 and p=0.323. In our study, the correlation between mean arterial pressure and photoplethysmography ranged from 0.2 to 0.3, indicating a weak association. Thus, flap perfusion is not strongly dependent on SBP under normovolemic conditions. The results of the Spearman correlation showed no statistically significant association between average value of three days PPG value and either mean arterial pressure [r_s_=0.31, p=0.131] or systolic blood pressure [r_s_=0.11, p=0.595]. 

**Figure 6:**
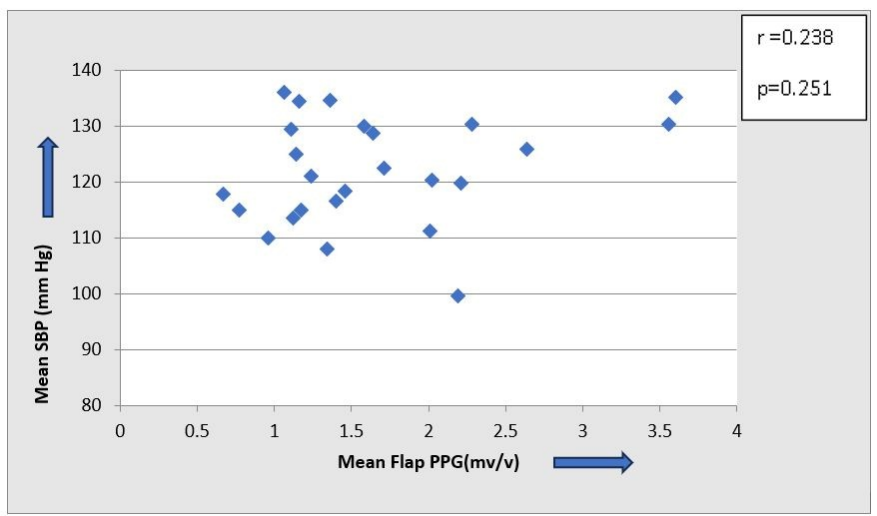
Scatter plot to show correlation between systemic blood pressure and flap

**Figure 7:**
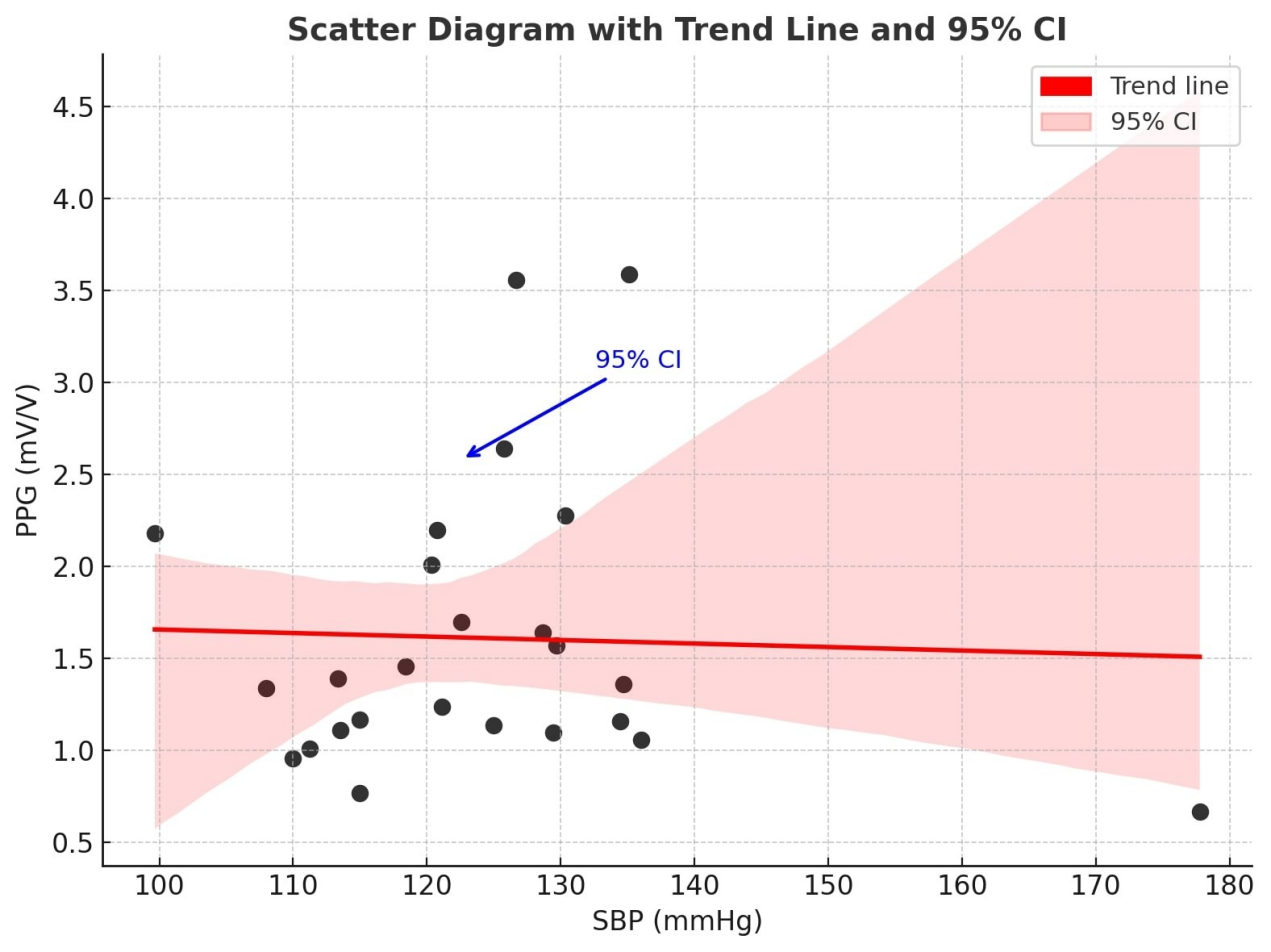
Scatter plot with a trend line plus 95% CI

The scatter plot of the overall mean systolic blood pressure and perfusion of the flap reveals a scattered distribution of the variables with no apparent trend or correlation, suggesting that blood pressure and perfusion are independent variables (Figure 6 (Fig. 6), Figure 7 (Fig. 7)). 

## Discussion

Microsurgical procedures are now routinely performed thanks to the availability of specialized instruments including operating loupes, microscopes, superfine microsurgery instruments, venous couplers, and micro sutures. With training and technical refinement, the failure rate has declined to 2–3% [8]. Instrumentation and technical factors no longer determine the success of microsurgery in the majority of cases. 

Preoperative immutable risk factors that may increase the risk of free flap failure are age, smoking, arterial hypertension, peripheral arterial disease, diabetes mellitus, and coronary artery disease [9]. Other factors are the location of the defect and etiology. Diabetes mellitus also increases the risk of flap failure 2.5-fold [10]. 

Intraoperatively, the choice of anesthetic and type and quantity of fluids used also determines the success of free tissue transfer. Blood pressure, use of vasoactive drugs, and length of the operation are other important factors in the outcome of free flap surgery. 

There is always concern in the mind of the reconstructive surgeon regarding maintaining adequate blood pressure for optimal perfusion of flaps. Free flaps are tissues whose all neural connections have been severed [11]. Assuming that there are no autonomic connections; after completing the anastomosis, the blood perfusion to the flap can be a direct function of blood pressure. Thus, this study explored how blood pressure affects perfusion of the free flap. To avoid the confounding variables, we selected patients without any comorbidities. MAP below 65 mm Hg was considered hypotensive. In our series, none of the cases were hypotensive postoperatively. All patients were administered fluid as per the output. For 3 days postoperative, patients were given fluid by intravenous or nasogastric as per their indication of surgery.

In 2016, Nilaman et al. [11] identified various preoperative factors that affect the perfusion of the flap. According to the Poiseuille equation 
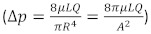
, they concluded, considering the length of the vessel (L) fixed, perfusion of the flap can be affected by the pressure gradient (mainly determined by the systolic arterial pressure), radius of the vessel (r), and viscosity of blood (µ). Thus, the external factor that can be controlled is the systolic arterial pressure [11]. To maintain adequate systolic arterial pressure and perfusion of the flap, two factors can be regulated: IV fluids and vasopressors. The common intellect of the reconstructive surgeon is to keep the blood pressure on higher side for adequate perfusion of the free flap postoperatively. Though no studies have correlated blood pressure and perfusion of flaps, some have analyzed the role of intraoperative blood pressure in the flap outcome. 

In a retrospective analysis of 46 free fibula flaps in head and neck reconstruction, no direct correlation was found between flap-related complications like dehiscence, flap necrosis, anastomotic insufficiency, or thrombosis between intraoperative or immediate postoperative short term blood pressure fluctuations. The MAP was measured intraoperatively and 24 hours postoperatively. However, the duration of the fluctuations is not mentioned. In our study, the cumulative mean of mean arterial pressure of all patients of three days monitoring was 91.26 ± 5.83 mm Hg. The mean correlation coefficient (*r*) was 0.206, with a *p*-value of 0.323, when compared with the overall mean flap PPG values across all three days. Given that all patients in our study were normovolemic clinically without any vasopressors, MAP, therefore, has no effect on the baseline perfusion of free flaps [12]. These findings suggest that aggressive elevation of blood pressure does not translate into improved flap perfusion. Clinically, it is reasonable to target a MAP of approximately 65 mm Hg, promptly correct hypotension with fluids or vasopressors when required, and avoid unnecessary fluid loading undertaken solely to increase blood pressure, as this may contribute to complications without enhancing perfusion.

A retrospective analysis of 445 cases of free flap in head–neck reconstruction was conducted by Kass et al. [13]. To standardize blood pressure data, MAP and fractional change in MAP (FCM) were used. Regression analysis concluded MAP <60 mm Hg as hypotension and FCM >20% the lability. In their series of 445 free flaps, 35 cases resulted in flap loss. The only hemodynamic variable that showed a significant association with these losses was a MAP <60 mm Hg, which exhibited a clear dose-response relationship. Additionally, FCM (isolated blood pressure lability) was not associated with flap loss. In our series, the overall mean systolic blood pressure, *r* (Pearson’s correlation coefficient), and flap PPG value for 3 days were 121.94 mm Hg, 0.238, and 1.65, respectively. Systolic blood pressure was not found associated with flap PPG value. Thus, these findings highlight an autoregulatory plateau, wherein microvascular flow stabilizes under normovolumic conditions after the perfusion pressure surpasses a threshold of approximately 60 mm Hg (MAP) [13].

Contrary to common belief that maintaining hyperdynamic fluid volume is important, excess fluid may be deleterious. Based on published data, some common beliefs need to be cleared based on the published data. Crystalloid infusion of more than 130 mL/kg/hr. poses separate risks factors and predictor of flap failure [14]. It has been suggested that increased fluid may lead to excessive flaps or recipient side edema. This may not only lead to stress on the pedicle but also impair the healing of the suture lines [15]. 

Similarly, the use of vasopressors perioperatively is thought to compromise flap vascularity. Yet, another study concluded that use of vasopressors intraoperatively does not increase the incidence of free flap failure. By studying individual agents intraoperatively using laser Doppler velocimetry, they showed that epinephrine/dopexamine decreases whereas norepinephrine/dobutamine increases free flap skin blood flow [16]. 

It has been suggested that hypervolemia and maintenance of high blood pressure levels are not required for maintaining adequate perfusion. Other studies indicate that intraoperative and postoperative blood pressure fluctuations are common and do not affect flap perfusion. Earlier studies have set MAP at 60 mm Hg or long periods of hypotension below which the vascularity of flap may be compromised. Our study concludes that perfusion of the free flap is not a direct function of blood pressure in clinically normovolemic patients. 

The present study is limited by a small sample size and the inclusion of different types of flaps, which precludes meaningful subgroup analysis. Furthermore, the study cohort comprised patients within a restricted blood pressure range, limited to normovolemic individuals with a mean arterial pressure above 65 mm Hg and without hypertension. While PPG using optical devices represents a sensitive method for detecting tissue perfusion, its accuracy may be compromised by factors such as suboptimal signal quality, inadequate surface contact, and device-specific calibration requirements. A significant relationship may be established with a larger sample size.

## Conclusion

Above a critical level of MAP, the perfusion of the free flap does not directly correlate with postoperative blood pressure. Long episodes of hypotension and MAP <60 mm Hg may jeopardize blood supply and perfusion of the free flap.

## Notes

### Authors’ ORCIDs

- Shamendra Anand Sahu: 0000-0003-0877-8725
- Jiten Kumar Mishra: 0000-0001-6357-5299
- Moumita De: 0000-0001-5016-1636
- Jalaz Joezer Rahmi: 0000-0002-3330-0136
- Abhijith Valsalan: 0000-0001-9827-3645
- Ripu Daman Arora: 0000-0003-0522-3118

### Competing interests

The authors declare that they have no competing interests.

## Figures and Tables

**Table 1:**
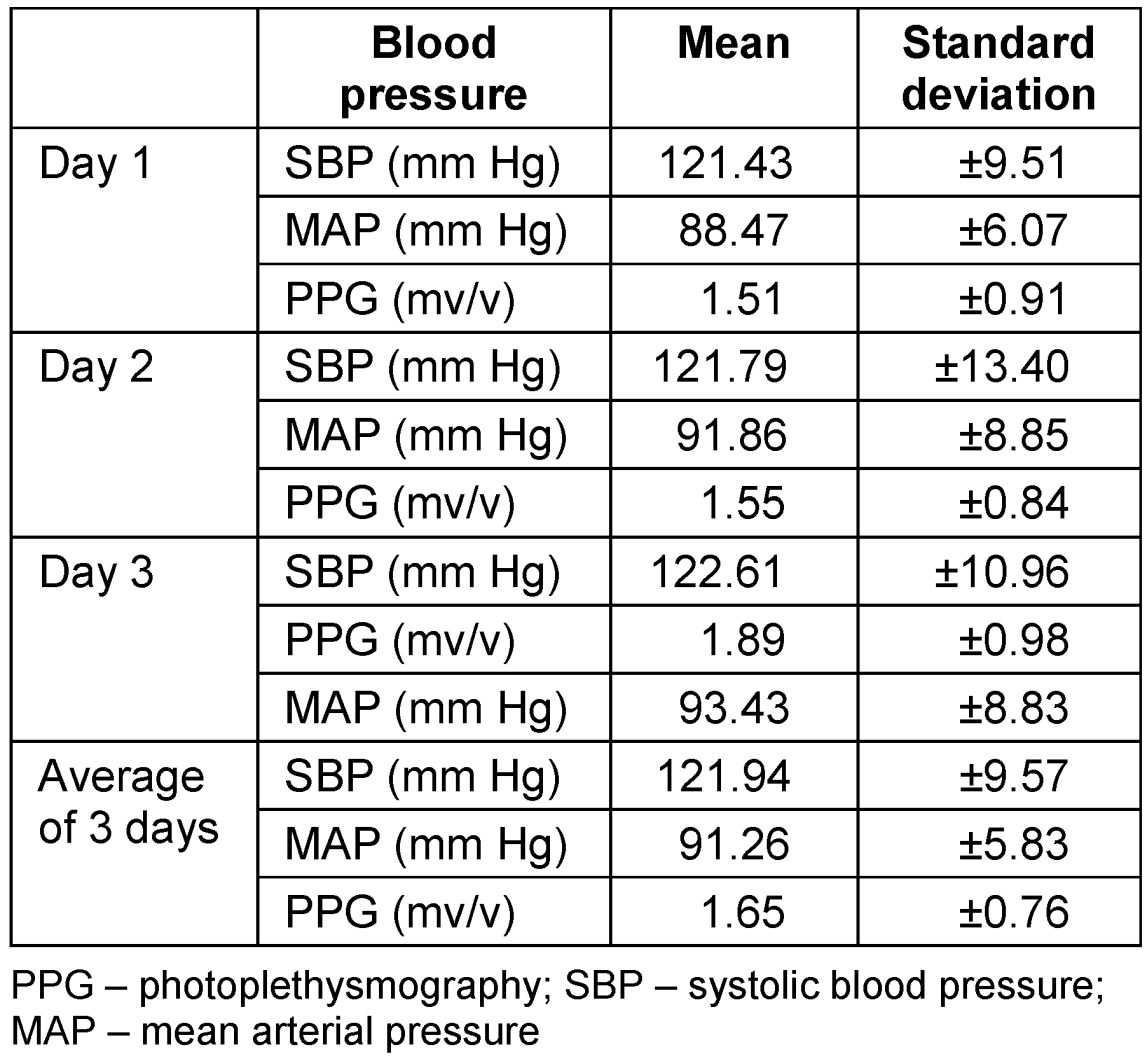
Cumulative values of blood pressure and photoplethysmography of all patients

**Table 2:**
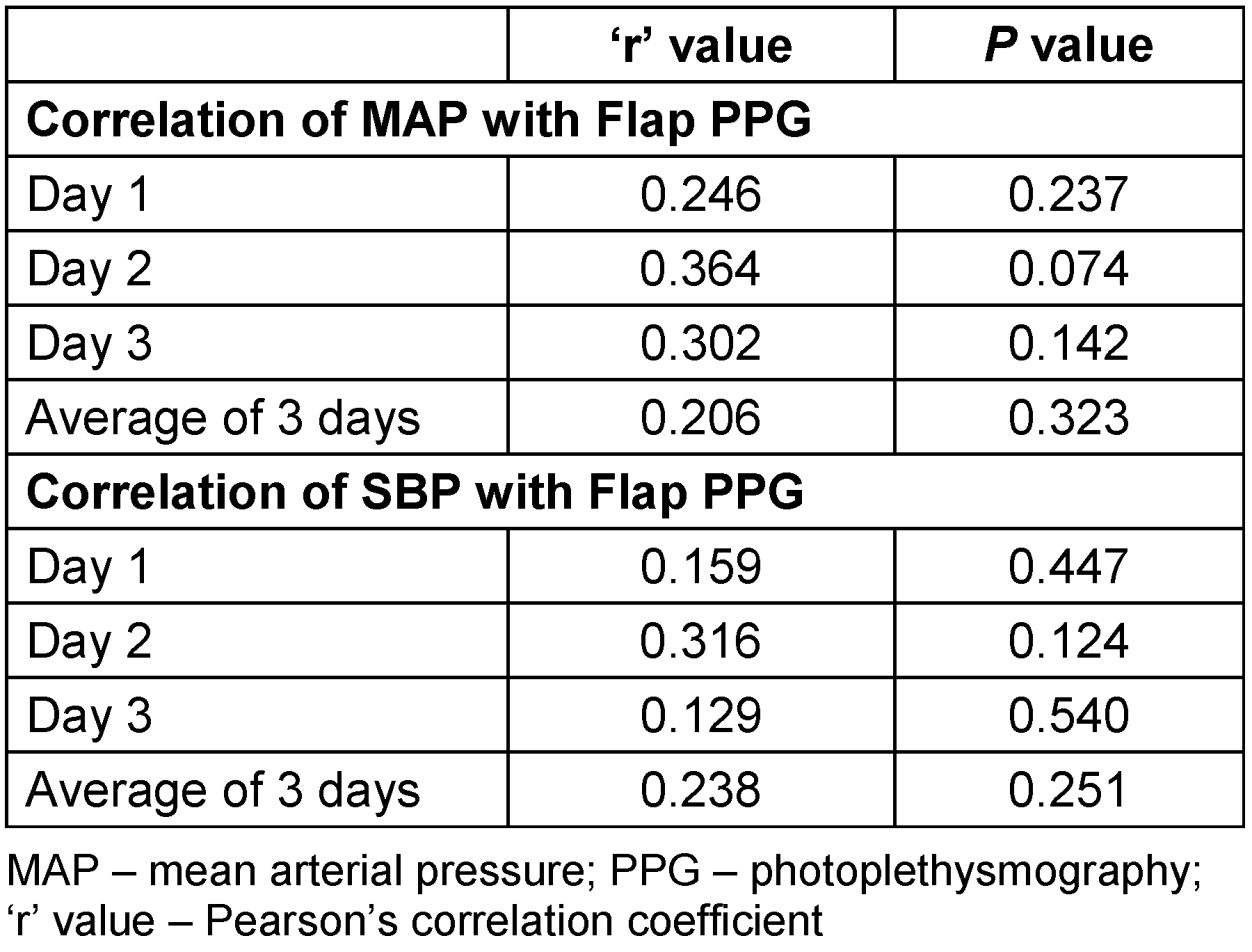
Statistical relationship between blood pressure and PPG value

**Table 3:**
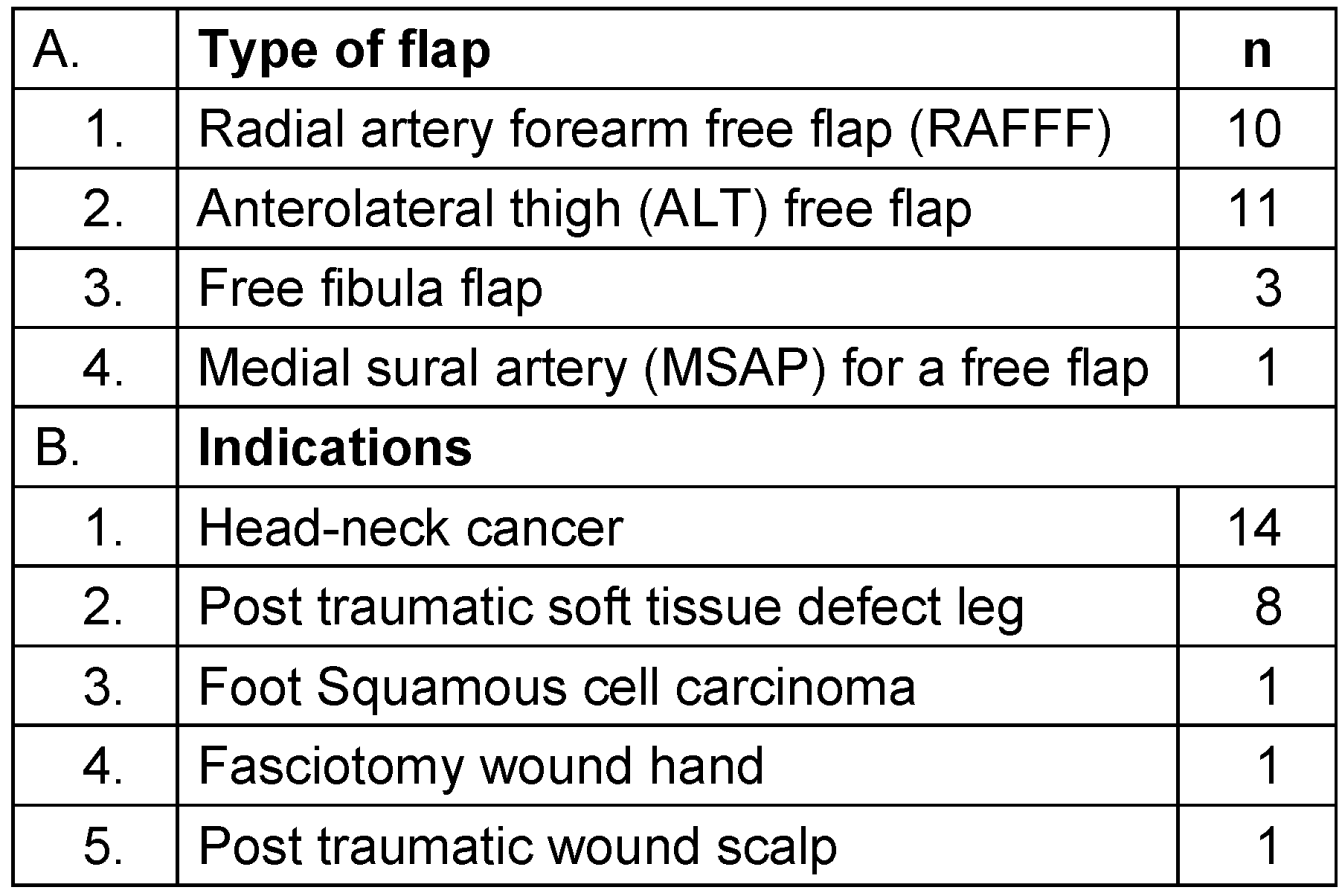
Indications and type of flap done
